# Gender, alexithymia and physical inactivity associated with abdominal obesity in type 1 diabetes mellitus: a cross sectional study at a secondary care hospital diabetes clinic

**DOI:** 10.1186/s40608-017-0157-1

**Published:** 2017-06-02

**Authors:** Eva O. Melin, Ralph Svensson, Maria Thunander, Magnus Hillman, Hans O. Thulesius, Mona Landin-Olsson

**Affiliations:** 1 Lund University, Faculty of Medicine, Department of Clinical Sciences, Lund, Sweden; 2 Department of Research and Development, Region Kronoberg, Box 1223, SE-351 12 Växjö, Sweden; 3 Primary Care, Region Kronoberg, Växjö, Sweden; 4 Department of Psychology, Linnaeus University, Växjö, Sweden; 5 Department of Internal Medicine, Central Hospital, Växjö, Sweden; 6 Department of Clinical Sciences, Diabetes Research Laboratory, Lund University, Faculty of Medicine, Lund, Sweden; 7 Department of Clinical Sciences, Division of Family Medicine, Lund University, Malmö, Sweden; 8 Department of Endocrinology, Lund University, Skane University Hospital, Lund, Sweden

**Keywords:** Alexithymia, Anxiety, Cardiovascular complications, Depression, Emotions, Gender, Obesity, Physical activity, Self-image, Type 1 diabetes mellitus

## Abstract

**Background:**

Obesity is linked to cardiovascular diseases and increasingly common in type 1 diabetes mellitus (T1DM) since the introduction of intensified insulin therapy. Our main aim was to explore associations between obesity and depression, anxiety, alexithymia and self-image measures and to control for lifestyle variables in a sample of persons with T1DM. Secondary aims were to explore associations between abdominal and general obesity and cardiovascular complications in T1DM.

**Methods:**

Cross sectional study of 284 persons with T1DM (age 18–59 years, men 56%), consecutively recruited from one secondary care hospital diabetes clinic in Sweden. Assessments were performed with self-report instruments (Hospital Anxiety and Depression Scale, Toronto Alexithymia Scale-20 items and Structural Analysis of Social Behavior). Anthropometrics and blood samples were collected for this study and supplemented with data from the patients’ medical records. Abdominal obesity was defined as waist circumference men/women (meters): ≥1.02/≥0.88, and general obesity as BMI ≥30 kg/m^2^ for both genders. Abdominal obesity was chosen in the analyses due to the high association with cardiovascular complications. Different explanatory logistic regression models were elaborated for the associations and calibrated and validated for goodness of fit with the data variables.

**Results:**

The prevalence of abdominal obesity was 49/284 (17%), men/women: 8%/29% (*P* < 0.001). Abdominal obesity was associated with women (AOR 4.9), physical inactivity (AOR 3.1), alexithymia (AOR 2.6) and age (per year) (AOR 1.04). One of the three alexithymia sub factors, “difficulty identifying feelings” (AOR 3.1), was associated with abdominal obesity. Gender analyses showed that abdominal obesity in men was associated with “difficulty identifying feelings” (AOR 7.7), and in women with use of antidepressants (AOR 4.3) and physical inactivity (AOR 3.6). Cardiovascular complications were associated with abdominal obesity (AOR 5.2).

**Conclusions:**

Alexithymia, particularly the alexithymia subfactor “difficulty identifying feelings”, physical inactivity, and women, as well as cardiovascular complications were associated with abdominal obesity. As abdominal obesity is detrimental in diabetes due to its association with cardiovascular complications, our results suggest two risk factor treatment targets: increased emotional awareness and increased physical activity.

## Background

Obesity, particularly abdominal obesity, is an important risk factor for impaired glycemic control, cardiometabolic diseases and increased mortality [1–4]. The prevalence of obesity is increasing in Sweden as well as globally [5, 6]. In Sweden in 2009, when this study was conducted, the prevalence of general obesity (BMI ≥ 30 kg/m^2^) was 11% in men, and 10% in women [7]. Traditionally in type 1 diabetes mellitus (T1DM), people were lean, the prevalence of the metabolic syndrome was low, and cardiovascular complications were rare [8]. Since the introduction of intensified insulin therapy in order to optimize glycemic control and to reduce microvascular complications, the prevalence of obesity, other features of the metabolic syndrome and cardiovascular complications have increased dramatically [8–10]. Cardiovascular disease is now a leading cause of death for persons with T1DM [8]. Particularly girls/women with T1DM are at risk for developing overweight and obesity [10].

Obesity has been associated with emotional eating, the tendency to eat when experiencing negative emotions [11, 12]. Emotional eating can be both a conscious behaviour to ease emotional distress, as well as an automatic reaction to unrecognized negative feelings i.e. reflexive emotional eating [13]. The latter has been associated with alexithymia [13], which is defined by difficulty identifying feelings, difficulty describing feelings, externally oriented thinking and constricted imaginative processes [14]. Persons with alexithymia have difficulties distinguishing bodily sensations due to emotional arousal from bodily sensations of somatic origin [14]. Alexithymia has been linked to obesity [15–17], and to cardiovascular mortality in men [18].

Persons with T1DM have an increased risk of depression [19]. The impact of depression on weight is depending on depression type [20, 21]. Melancholic depression is characterized by weight loss, whereas atypical depression is characterized by weight gain [20–22]. Whether either of these two depression types is more common in persons with T1DM is not known. Depression in diabetes have been linked to life style factors such as less physical activity and unhealthy diet [23]. We have previously shown that self-reported depression in this population was associated with impaired glycemic control [3] and increased midnight cortisol secretion [24]. Even if there is evidence for a positive association between obesity and anxiety disorders [25, 26], the strength of the association is depending on anxiety subtype [26]. There are gender differences, women are more affected by depression and anxiety than men, which unfortunately often go unrecognized and untreated [27].

Since persons with obesity often are stigmatized and face prejudice and discrimination [28, 29], this may negatively impact self-identity and self-perception [29]. Obese persons have indeed described self-hate, shame, guilt, and disgust directed to themselves [29].

Diabetes emotional distress is an emotional response to a demanding health-related condition in diabetes [30]. Failure to achieve diabetes treatment goals, such as maintaining or achieving normal weight, might induce diabetes emotional distress [30], which could potentially be expressed as self-blame or self-hate.

Our main hypotheses were that psychological factors are linked to obesity, and that increased knowledge of these links might be beneficial for the development of new strategies to target obesity in persons with T1DM. Our main aim was to explore associations between obesity and depression, anxiety, alexithymia and self-image in a sample of persons with T1DM controlling for potential confounders such as smoking and physical inactivity. Secondary aims were to explore associations between abdominal and general obesity and cardiovascular complications in T1DM.

## Methods

### Participants and procedures

**Fig. 1:**
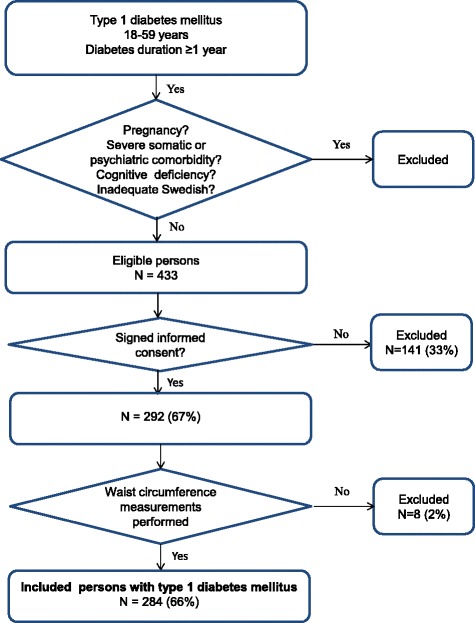
Description of criteria for inclusion in this study of obesity in persons with T1DM

This report has a cross sectional design and is one of three baseline analyses [3, 24] for a randomized controlled trial (ClinicalTrials.gov: NCT01714986) where “Affect School with Script Analysis” was tried against “Basic Body Awareness Therapy” for persons with diabetes, inadequate glycemic control and psychological symptoms [31, 32]. The participants were outpatients, recruited from one secondary care hospital diabetes clinic with a catchment population of 125,000 in southern Sweden during, the period 03/25/2009 to 12/28/2009. The participants were recruited consecutively by specialist diabetes physicians or diabetes nurses at regular follow up visits. In this study 284 persons with T1DM were included, 66% of the eligible patients, see Fig. 1. Somatic exclusion criteria were cancer, hepatic failure, end-stage renal disease, stroke with cognitive deficiency, or visual impairment to such a degree that reading the questionnaires was impossible. Mental exclusion criteria were psychotic disorder, bipolar disorder, severe personality disorder, severe substance abuse, or mental retardation. Patients underwent assessments with self-report instruments at the regular follow up visits; anthropometrics and blood samples were collected. Data regarding patients’ diagnoses, cardiovascular complications, life style factors, and medication were collected from computerized medical records.

### Self-report instruments

Self-reported depression and anxiety were assessed by the Hospital Anxiety and Depression Scale (HADS) [33]. The depression (HADS-D) and anxiety (HADS-A) subscales, consists of 7 statements each, with 4 response alternatives from 0 to 3. The cut off level ≥ 8 points, recommended by the constructors, was used for both subscales to define self-reported depression and anxiety [33]. To try the validity of HADS, clinical psychiatric diagnosis and antidepressants use were tried against self-reported depression and self-reported anxiety.

Alexithymia was assessed by the Toronto Alexithymia Scale-20 items (TAS-20) which consists of 20 statements rated from 1 to 5 [11, 16, 17, 34, 35]. The cut off level ≥ 61 points for TAS-20 total scores, recommended by the constructors, was used to define alexithymia [17, 34, 35]. TAS-20 is based on three subscales “difficulty identifying feelings” (DIF), “difficulty describing feelings” (DDF), and “externally oriented thinking” (EOT). No normal values are presented for the subscales by the constructors [34, 35]. All three subscales were dichotomized at the 90th percentile.

Self-image measures were assessed by the Structural Analysis of Social Behavior assessment tool (SASB) [36, 37]. The questionnaire comprises 36 self-referential statements with response options on a scale from 0 to 100 with 10-point increments. The results were summarized into eight subscales (clusters): 1) self-emancipation, 2) self-affirmation, 3) self-love, 4) self-protection, 5) self-control, 6) self-blame, 7) self-hate, and 8) self-neglect. No normal values are presented for the subscales by the constructors [37]. Subscales 1, 6, 7 and 8 were dichotomized at the 90th percentile. Subscales 2, 3, 4 and 5 were dichotomized at the 10th percentile.

### Anthropometrics

Waist circumference (WC), weight and length were measured by a nurse according to standard procedures. Abdominal obesity was defined as WC ≥ 1.02 m for men and as WC ≥ 0.88 m for women [1, 2, 4]. General obesity was defined as Body Mass Index (BMI) ≥ 30 kg/m^2^ for both genders [2].

### Blood analyses

Venous HbA1c was analyzed with high pressure liquid chromatography, HPLC - variant II, Turbo analyzer (Bio – Rad®, Hercules, CA, USA) [38], at the department of Clinical Chemistry, Växjö Central Hospital. High HbA1c was defined as DCCT >8.6% (IFCC >70 mmol/mol) [3].

### Data collection from medical records

Data regarding life style variables were collected. Smokers were defined as having smoked any amount of tobacco during the last year. Physical inactivity was defined as moderate activities, such as 30 min of walking, less than once a week.

Clinical psychiatric diagnoses were established prior to recruitment, and were dichotomized as having or not having a psychiatric diagnosis. Use of antidepressants was dichotomized as using or not using any type of antidepressant.

Diabetes specific treatment was divided into three groups: Multiple daily insulin injections (MDII); continuous subcutaneous insulin infusion (CSII); and MDII combined with oral antidiabetic agents (OAA).

Cardiovascular complications were defined as ischemic heart disease, stroke or transient ischemic attack.

### Statistical analysis

Analysis of data distribution using histograms revealed that the test results for the self-report instruments, age and diabetes duration were not normally distributed. Data were presented as median (quartile (q)_1_, q_3_; min-max), or as median (10th, 90th percentile; min-max) and analyses were performed with Mann-Whitney *U* test. Fisher’s exact test (two-tailed) was used to analyze categorical data. A backward elimination multiple logistic regression analysis with cardiovascular complications as dependent variable showed that abdominal obesity had a higher association than general obesity. Thus, abdominal obesity was chosen in subsequent multiple regression modelling. Different explanatory logistic regression models were elaborated for the associations, and calibrated and validated for goodness of fit with the data variables. First crude odds ratios (CORs) were calculated. Then variables with *P* ≤ 0.10 were entered into multiple logistic regression models (Backward: Wald). The Hosmer and Lemeshow test for goodness-of-fit and Nagelkerke R^2^ were used to evaluate and calibrate the models. Confidence intervals (CIs) of 95% were used. *P* < 0.05 was considered statistically significant. SPSS® version 18 (IBM, Chicago, Illinois, USA) was used for all statistical analyses.

## Results

### Baseline data and test results for all and gender specified

In this study of obesity and psychological states and traits in TIDM (*n* = 284, age 18–59 years, men 56%), persons with abdominal obesity (*n* (%) = 49 (17)) were compared with persons without abdominal obesity (*n* (%) = 235 (83)). Baseline data are presented in Table 1. The women had 3.6 times higher prevalence of abdominal obesity than the men (29% compared to 8%, *P* < 0.001). None of the participants had been diagnosed with an eating disorder, according to their medical records.

**Table 1:** Baseline characteristics for the 284 patients with T1DM presented for all and gender specified

	All	Men	Women	*P* ^*a*^
*N*	284	159	125	
Age (years)	42 (32, 51; 18–59)	43 (32, 52)	41 (30, 50)	0.12^b^
Diabetes duration (years)	20 (11, 30; 1–55)	21 (11–32)	19 (11, 29)	0.26^b^
Abdominal obesity^c^	49 (17)	13 (8)	36 (29)	<0.001
General obesity^d^	34 (12)	11 (7)	23 (18)	0.005
High HbA1c^e^	78 (28)	39 (24)	39 (31)	0.23
Smoking ^f^	28 (10)	18 (12)	10 (8)	0.42
Physical inactivity^g^	31 (11)	18 (12)	13 (11)	0.85
Cardiovascular complications	10 (4)	6 (4)	4 (3)	>0.99
Clinical psychiatric diagnoses	38 (13)	16 (10)	22 (17)	0.079
Antidepressants	23 (8)	11 (7)	12 (10)	0.51
MDII^h^ and OAA^i^	17 (6)	6 (4)	11 (9)	0.15
CSII^j^	26 (9)	13 (8)	13 (10)	
MDII^h^	241 (85)	140 (88)	101 (81)	

Data are *n* (%) or median (q_1_, q_3_; min-max)

^a^Fisher’s exact test unless otherwise specified

^b^Mann-Whitney U test

^c^Abdominal obesity: WC men/women (meters): ≥ 1.02/≥ 0.88

^d^General obesity: BMI ≥ 30 kg/m^2^

^e^High HbA1c: >8.6% (>70 mmol/mol)

Missing values men/women: ^f^ *n* = 6/6; ^g^ *n* = 6/6

^h^Multiple daily insulin injections

^i^Oral antidiabetic agents. ^j^ Continuous subcutaneous insulin infusion

The test results for the self-report instruments are presented in Table 2. Women had higher prevalence of self-reported anxiety (*P* < 0.001), high self- blame (*P* < 0.001), high self-hate (*P* = 0.001), low self-affirmation (*P* = 0.001), low self-love (*P* = 0.020), and high DIF (*P =* 0.036). Men had higher prevalence of high EOT (*P* < 0.035).

**Table 2:** Test results for HADS, TAS-20 and SASB, for the 284 patients with T1DM presented for all and gender specified

	Scores ^a^	Prevalence^b^			
	All	All	Men	Women	*P* ^*c*^
*N*	284	284	159	125	
HADS					
HADS-D scores/Depression (≥ 8p)	3 (1, 8; 0–20)	29 (10)	15 (9)	14 (11)	0.70
HADS-A scores/Anxiety (≥ 8p)	5 (1, 11; 0–18)	86 (34)	39 (24)	57 (46)	<0.001
TAS-20					
TAS-20 scores/Alexithymia (≥ 61p)	46 (32, 64; 20–81)	42 (15)	19 (12)	23 (18)	0.13
DIF scores/high DIF (≥ 23 p)	13 (8, 23; 7–31)	32 (11)	12 (8)	20 (16)	0.036
DDF scores /high DDF (≥ 18 p)	12 (6, 18; 5–32)	30 (11)	17 (11)	13 (10)	>0.99
EOT scores /high EOT (≥ 27 p)	21 (13, 27; 8–33)	31 (11)	23 (14)	8 (6)	0.035
SASB^d^					
Self-emancipation scores /high self-emancipation (≥ 66 p)^d^	50 (34, 66; 8–94)	27 (10)	18 (12)	9 (7)	0.31
Self-affirmation scores /low self-affirmation (≤ 38 p)^d^	70 (38, 88; 8–100)	32 (12)	9 (6)	23 (19)	0.001
Self-love scores /low self-love (≤ 40 p)^d^	64 (40, 86; 14–100)	31 (11)	11 (7)	20 (16)	0.020
Self-protection scores /low self-protection (≤ 30 p)^d^	52 (30, 75; 5–95)	28 (10)	19 (12)	9 (7)	0.23
Self-control scores /low self-control (≤ 28 p)^d^	52 (28, 74; 0–98)	33 (12)	18 (12)	15 (12)	0.85
Self-blame scores /high self-blame (≥ 52 p)^d^	18 (0, 52; 0–95)	37 (13)	9 (6)	28 (23)	<0.001
Self-hate scores /high self-hate (≥ 50 p)^d^	16 (0, 50; 0–92)	30 (11)	8 (5)	22 (18)	0.001
Self-neglect scores /high self-neglect (≥ 48 p)^d^	22 (5, 48; 0–80)	31 (11)	18 (11)	13 (10)	0.85

^a^Data are median (10th, 90th percentile; min-max)

^b^ *N* (%)

^c^Fisher’s exact test. ^d^Missing values men/women: *n* = 3/4

### Cardiovascular complications and obesity

Cardiovascular complications were associated with abdominal obesity (adjusted odds ratio (AOR) (CI) 5.2 (1.5–18.8), *P* = 0.011), whereas general obesity was not (AOR (CI) 0.9 (0.2–5.7), *P* = 0.95).

### Comparisons between persons with and without abdominal obesity

The results of the comparisons are presented in Table 3. The 49 obese persons compared to the 235 non-obese had higher prevalence of high DIF (*P* = 0.005), self-reported anxiety (*P* = 0.007), cardiovascular complications (*P* = 0.016), high self-hate (*P* = 0.021), use of antidepressants (*P* = 0.038), and alexithymia (*P* = 0.046). The 13 obese men had higher prevalence of high DIF (*P* = 0.009) than the 146 non-obese men. The 36 obese women had higher prevalence of cardiovascular complications (*P* = 0.006) and use of antidepressants (*P* = 0.038) than the 89 non-obese women.

**Table 3:** Comparisons between T1DM patients with and without abdominal obesity for all and gender specified

	All patients			Men			Women		
	Obesity^a^	Non-obesity	*P* ^a^	Obesity^a^	Non-obesity	*P* ^a^	Obesity^a^	Non-obesity	*P* ^a^
N	49	235		13	146		36	89	
Age (years)	45 (35, 53)	42 (31, 51)	0.11^b^	50 (34, 58)	43 (32, 51)	0.072 ^b^	45 (35–51)	40 (30–48)	0.18^b^
Diabetes duration (years)	22 (14, 28)	20 (11, 31)	0.64^b^	21 (8, 31)	20 (12, 32)	0.92 ^b^	23 (15–29)	18 (10–29)	0.27^b^
Depression (≥ 8p)^c^	6 (12)	23 (10)	0.61	2 (15)	13 (9)	0.35	4 (11)	10 (11)	>0.99
Anxiety (≥ 8p)^d^	25 (51)	71 (30)	0.007	3 (31)	35 (24)	0.74	21 (58)	36 (40)	0.078
Alexithymia (≥ 61p)^e^	12 (24)	30 (13)	0.046	3 (23)	16 (11)	0.19	9 (25)	14 (16)	0.31
High DIF (≥23 p)^e^	12 (24)	20 (8)	0.005	4 (31)	8 (6)	0.009	8 (22)	12 (14)	0.28
High DDF (≥ 18 p)^e^	4 (8)	26 (11)	0.80	1 (8)	16 (11)	>0.99	3 (8)	10 (11)	0.72
High EOT (≥ 27 p)^e^	2 (4)	29 (12)	0.13	0 (0)	23 (16)	0.22	2 (6)	6 (7)	>0.99
High self-emancipation (≥ 66 p)^f^	6 (12)	21 (9)	0.43	3 (23)	15 (10)	0.18	3 (9)	6 (7)	0.72
Low self-affirmation (≤ 38 p)^f^	9 (19)	23 (10)	0.13	1 (8)	8 (6)	0.55	8 (23)	15 (17)	0.61
Low self-love (≤ 40 p)^f^	8 (17)	23 (10)	0.21	2 (15)	9 (6)	0.23	6 (17)	14 (16)	>0.99
Low self-protection (≤ 30 p)^f^	5 (10)	23 (10)	>0.99	1 (8)	18 (13)	>0.99	4 (11)	5 (6)	0.28
Low self-control (≤ 28 p)^f^	8 (17)	25 (11)	0.32	2 (15)	16 (11)	0.65	6 (17)	9 (10)	0.36
High self-blame (≥ 52 p)^f^	9 (19)	28 (12)	0.24	1 (8)	8 (6)	0.55	8 (23)	20 (23)	>0.99
High self-hate (≥ 50 p)^f^	10 (21)	20 (9)	0.021	1 (8)	7 (5)	0.51	9 (26)	13 (15)	0.20
High self-neglect (≥ 48 p)^f^	7 (14)	24 (10)	0.45	1 (8)	17 (12)	>0.99	6 (17)	7 (8)	0.19
Smoking^g^	4 (9)	24 (11)	0.80	1 (8)	17 (12)	>0.99	3 (9)	7 (8)	>0.99
Physical inactivity^h^	9 (19)	22 (10)	0.084	2 (15)	16 (11)	0.65	7 (20)	6 (7)	0.054
Cardiovascular complications	5 (10)	5 (2)	0.016	1 (8)	5 (3)	0.41	4 (11)	0	0.006
Clinical psychiatric diagnoses	11 (22)	27 (11)	0.062	1 (8)	15 (10)	>0.99	10 (28)	12 (14)	0.071
Antidepressants	8 (16)	15 (6)	0.038	1 (8)	10 (7)	>0.99	7 (19)	5 (6)	0.038
MDII and OAA	13 (27)	4 (2)	<0.001	3 (23)	3 (2)	0.009	10 (28)	1 (1)	<0.001
CSII	1 (2)	25 (10)		0	13 (9)		1 (3)	12 (14)	
MDII	35 (71)	206 (88)		10 (77)	130 (89)		25 (69)	76 (85)	

Obesity defined as men/women: WC ≥ 1.02/≥ 0.88 m. Data are *n* (%) or median (q_1_, q_3_)

^a^Fisher’s exact test unless otherwise indicated

^b^Mann-Whitney *U* test

^c^HADS-D

^d^HADS-A

^e^TAS-20

^f^SASB

Missing values for obese/non-obese men: ^f^ *n* = 0/3

^g^ *n* = 0/6

^h^ *n* = 0/6. Missing values for obese/non-obese women. ^f^ *n* = 1/3; ^g^ *n* = 2/4; ^h^ *n* = 1/5

### Associations between psychological states and traits, life style variables and abdominal obesity

In Table 4 variables associated with abdominal obesity are presented in two models for all patients. In model 1, abdominal obesity was associated with women (AOR 4.9), physical inactivity (AOR 3.1), alexithymia (AOR 2.6), and age (per year) (AOR 1.04). In model 2, abdominal obesity was associated with women (AOR 4.9), physical inactivity (AOR 3.5), high DIF (AOR 3.1), and age (per year) (AOR 1.04). Treatment with the combination of MDII and OAA was strongly associated with obesity (COR 19.1).

**Table 4:** Associations between abdominal obesity and psychological, somatic and life style variables in the T1DM patients

	Abdominal obesity (*N* = 265)					
			Model 1		Model 2	
	COR	*P*	AOR	*P* ^a^	AOR	*P* ^a^
Gender (women)	4.5 (2.3–9.0)	<0.001	5.0 (2.4–10.3)	<0.001	4.9 (2.4–10.2)	<0.001
Depression (≥ 8p)^b^	1.3 (0.5–3.3)	0.61	-	-	-	-
Anxiety (≥ 8p)^c^	2.4 (1.3–4.5)	0.006	1.5 (0.7–3.1)	0.27	1.3 (0.6–2.8)	0.51
Alexithymia (≥ 61p)^d^	2.2 (1.0–4.7)	0.039	2.6 (1.1–6.0)	0.028	-	-
High DIF (≥23 p)^d^	3.5 (1.6–7.7)	0.002	-	-	3.1 (1.3–7.5)	0.011
High DDF (≥18 p)^d^	0.7 (0.2–2.1)	0.55	-	-	-	-
High EOT (≥ 27 p)^d^	0.3 (0.1–1.3)	0.11	-	-	-	-
High self-emancipation (≥66 p)^e^	1.4 (0.5–3.7)	0.48	-	-	-	-
Low self-affirmation (≤38 p)^e^	2.1 (0.9–4.8)	0.091	1.0 (0.3–3.0)	0.95	0.9 (0.3–2.6)	0.85
Low self-love (≤40 p)^e^	1.8 (0.7–4.3)	0.19	-	-	-	-
Low self-protection (≤30 p)^e^	1.0 (0.4–2.9)	0.94	-	-	-	-
Low self-control (≤28 p)^e^	1.6 (0.7–3.9)	0.27	-	-	-	-
High self-blame (≥52 p)^e^	1.7 (0.7–3.8)	0.23	-	-	-	-
High self-hate (≥50 p)^e^	2.8 (1.2–6.3)	0.017	1.1 (0.4–3.1)	0.80	1.1 (0.4–3.5)	0.85
High self-neglect (≥48 p)^e^	1.5 (0.6–3.6)	0.41	-	-	-	-
Smoking^f^	0.8 (0.3–2.4)	0.66	-	-	-	-
Physical inactivity^g^	2.1 (0.9–4.9)	0.083	3.1 (1.2–8.1)	0.023	3.5 (1.3–9.1)	0.012
Age (per year)	1.02 (1.00–1.05)	0.11	1.04 (1.00–1.07)	0.024	1.04 (1.00–1.07)	0.024
Diabetes duration	1.00 (0.98–1.03)	0.77	-	-	-	-
Cardiovascular complications	5.2 (1.5–18.9)	0.011	-	-	-	-
Clinical psychiatric diagnoses	2.2 (1.0–4.9)	0.044	0.9 (0.2–3.6)	0.86	0.9 (0.2–3.8)	0.93
Antidepressants	2.9 (1.1–7.2)	0.025	1.6 (0.6–4.7)	0.38	1.6 (0.6–4.7)	0.38
MDII and OAA	19.1 (5.9–62.0)	0.001	-	-	-	-
CSII	0.2 (0.03–1.8)	0.16	-	-	-	-
MDII	1		-	-	-	-

*N* = 284 unless otherwise specified

^a^ Multiple logistic regression analysis (Backward: Wald)

^b^HADS-D

^c^HADS-A

^d^TAS-20

^e^SASB

Missing values: ^e^ *n* = 7

^f^ *n* = 12

^g^ *n* = 12. Model 1/model 2: Hosmer and Lemeshow Test 0.685/0.735; Nagelkerke R Square 0.192/0.20

Variables associated with abdominal obesity are presented for each gender in Table 5. In men, high DIF (AOR 7.7) was associated with abdominal obesity. In women, use of antidepressants (AOR 4.3) and physical inactivity (AOR 3.6) were associated with abdominal obesity.

**Table 5:** Associations between abdominal obesity and psychological and life style variables presented for each gender

	Abdominal obesity in men (*N* = 159)				Abdominal obesity in women (*N* = 115)			
	COR	*P*	AOR	*P* ^a^	COR	*P*	AOR	*P* ^a^
Depression (≥ 8p) ^b^	1.9 (0.4–9.3)	0.45	-	-	0.99 (0.29–3.4)	0.98	-	-
Anxiety (≥ 8p) ^b^	1.4 (0.4–4.9)	0.51	-	-	2.1 (0.9–4.5)	0.071	1.7 (0.8–4.0)	0.20
Alexithymia (≥ 61p) ^c^	2.4 (0.6–9.8)	0.21	-	-	1.8 (0.7–4.6)	0.23	-	-
High DIF (≥23 p) ^c^	7.7 (1.9–30.4)	0.004	7.7 (1.9–30.4)	0.004	1.8 (0.7–5.0)	0.23	-	-
High DDF (≥18 p) ^c^	0.7 (0.1–5.6)	0.72	-	-	0.7 (0.2–2.8)	0.63	-	-
High EOT (≥ 27 p) ^c^	0.00	>0.99	-	-	0.8 (0.2–4.2)	0.81	-	-
High self-emancipation (≥66 p) ^d^	2.6 (0.6–10.3)	0.19	-	-	1.2 (0.3–5.3)	0.76	-	-
Low self-affirmation (≤38 p) ^d^	1.4 (0.2–12.2)	0.76	-	-	1.4 (0.5–3.7)	0.49	-	-
Low self-love (≤40 p) ^d^	2.7 (0.5–14.1)	0.24	-	-	1.1 (0.4–3.0)	0.91	-	-
Low self-protection (≤30 p) ^d^	0.6 (0.1–4,7)	0.61	-	-	2.1 (0.5–8.3)	0.29	-	-
Low self-control (≤28 p) ^d^	1.4 (0.3–7.1)	0.65	-	-	1.8 (0.6–5.4)	0.32	-	-
High self-blame (≥52 p) ^d^	1.4 (0.2–12.2)	0.76	-	-	1.0 (0.4–2.5)	0.96	-	-
High self-hate (≥50 p) ^d^	1.7 (0.2–14.3)	0.66	-	-	1.9 (0.7–5.1)	0.17	-	-
High self-neglect (≥48 p) ^d^	0.6 (0.1–5.2)	0.67	-	-	2.3 (0.7–7.5)	0.15	-	-
Smoking ^e^	0.6 (0.1–4.9)	0.64	-	-	1.1 (0.3–4.4)	0.92	-	-
Physical inactivity ^f^	1.4 (0.3–6.9)	0.67	-	-	3.2 (1.0–10.5)	0.049	3.6 (1.1–11.8)	0.037
Clinical psychiatric diagnoses	0.7 (0.1–6.0)	0.77	-	-	2.5 (1.0–6.4)	0.062	1.5 (0.4–6-7)	0.57
Age (per year)	1.05 (0.99–1.11)	0.091	1.04 (0.98–1.10)	0.19	1.02 (0.99–1.06)	0.19	1.02 (0.99–1.06)	0.21
Antidepressants	1.1 (0.1–9.6)	0.91	-	-	4.1 (1.2–13.8)	0.025	4.3 (1.2–15.0)	0.022

*N* (men/women) = 159/125 unless otherwise specified

^a^Multiple logistic regression analysis (Backward: Wald)

^b^HADS-D

^c^HADS-A

^d^TAS-20

^e^SASB

Missing values for men: ^d^ *n* = 3

^e^ *n* = 6

^f^ *n* = 6. Missing values for women

^d^ *n* = 4

^e^ *n* = 6

^f^ *n* = 6. Men/women: Hosmer and Lemeshow Test 0.226/0.910; Nagelkerke R Square 0.10/0.105

### Variables associated with women, alexithymia, DIF and physical inactivity - interaction tests

Associations with women: abdominal obesity (AOR 4.2 (2.0–8.7), *P* < 0.001), self-hate (AOR 3.1 (1.2–8.1), *P* = 0.022), age (per year) (AOR 0.98 (0.96–1.00), *P* = 0.046), and self-reported anxiety (AOR 1.8 (1.0–3.2), *P* < 0.050); and the *p*-values were higher than 0.36 for alexithymia, physical inactivity, low self-affirmation, physical inactivity, clinical psychiatric diagnosis, and use of antidepressants.

Associations with alexithymia: self-reported anxiety (AOR 3.6 (1.7–7.4), *P =* 0.001) and abdominal obesity (AOR 2.2 (1.0–4.9), *P* = 0.055); and the *p*-values were higher than 0.10 for physical inactivity, gender, low self-affirmation, high self-hate, physical inactivity, clinical psychiatric diagnosis, use of antidepressants, and age.

Associations with high DIF: self-reported anxiety (AOR 9.1 (3.2–26.0), *P* < 0.001), low self-affirmation (AOR 2.7 (1.0–7.0), *P* = 0.47) and abdominal obesity (AOR 2.5 (1.0–6.1), *P* = 0.055); the *p*-values were higher than 0.24 for physical inactivity, gender, high self-hate, physical inactivity, clinical psychiatric diagnosis, use of antidepressants, and age.

**Fig. 2:**
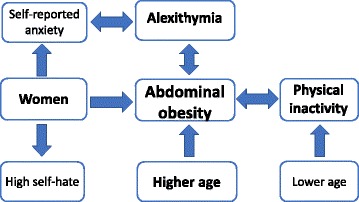
Illustration of significant associations found in this study

Associations with physical inactivity: abdominal obesity (AOR 2.6 (1.1–6.3), *P* = 0.034) and age (per year) (AOR 0.95 (0.92–0.99), *P* = 0.006); and the *p*-values were higher than 0.13 for alexithymia, gender, self-reported anxiety, low self-affirmation, high self-hate, clinical psychiatric diagnosis, and use of antidepressants. Significant associations with abdominal obesity, women, alexithymia and physical inactivity are presented in Fig. 2.

### Validation of HADS

Clinical psychiatric diagnosis was associated with self-reported depression (COR 10.8 (4.6–25.2), *P* < 0.001) and self-reported anxiety (COR 2.8 (1.4–5.6), *P* = 0.003). Antidepressants were associated with self-reported depression (COR 9.8 (3.8–25.3), *P* < 0.001) and self-reported anxiety (COR 3.4 (1.4–8.2), *P* = 0.006).

## Discussion

In this cross-sectional study of a population sample of 284 persons with T1DM, age 18–59 years, consecutively recruited from one hospital diabetes outpatient clinic, we found that female gender, physical inactivity and alexithymia were independently associated with abdominal obesity. The women had nearly 4 times higher prevalence of abdominal obesity than the men. High level of the alexithymia subfactor “difficulty identifying feelings” was responsible for the association between alexithymia and abdominal obesity. Gender analyses showed clear differences. In men, a high level of “difficulty identifying feelings” was strongly linked to abdominal obesity. In women, physical inactivity and the use of antidepressants were linked to abdominal obesity. The prevalence of general obesity in the women with T1DM was almost twice as high as in women in the Swedish population. Cardiovascular complications were strongly associated with abdominal obesity.

Strengths of our study are first that we systematically investigated the psychological states and traits in a well-defined population of persons with T1DM. Second, we explored interactions between the included variables. For example, at a first sight self-reported anxiety seemed to be associated with abdominal obesity which it was not in the further analyses. Instead self-reported anxiety was associated with both alexithymia and women which in turn were associated with abdominal obesity. Third, when the associations with abdominal obesity were tried the results were congruent. Clinical psychiatric diagnosis was not associated with abdominal obesity. Excess negative emotions, expressed as self-reported depression, anxiety, high levels of self-blame, self-hate, or self-neglect, were not linked to abdominal obesity; neither were low levels of positive emotions, expressed as low self-affirmation, low self-love or low self-protection. Fourth, we had access to both waist circumference measures and BMI, and we could choose in the analyses to use abdominal obesity, which showed the highest association with cardiovascular complications. Fifth, we validated the self-report instrument for depression and anxiety. We found strong associations with clinical psychiatric diagnosis and the use of antidepressants for both subscales of the self-report instrument.

The main limitation in our study was the small number of obese persons, particularly when the gender sub analyses were performed. There are several possible type two errors. The prevalence of alexithymia and high DIF were higher in both women and men with abdominal obesity compared to the non-obese. Also, the prevalence of physical inactivity was higher in both men and women with abdominal obesity compared to the non-obese. Second, there are difficulties connected to using the SASB and TAS-20 subscales as there are no normal values presented by the designers of these self-report instruments. We chose to compare the most extreme test results with the remaining, and all subscales were therefore dichotomized either at the 10th or 90th percentile. A third limitation is that we did not include any self-report instrument assessing diabetes related emotional distress.

This study is to our knowledge the first to demonstrate a link between alexithymia, mainly the subfactor “difficulty identifying feelings”, and abdominal obesity in persons with T1DM. However, an increased prevalence of alexithymia in persons with obesity but without diabetes has been shown [11, 15, 16]. Whether the direction of the association between abdominal obesity and alexithymia goes one-way or two-way cannot be concluded by this cross-sectional study. Alexithymia could lead to obesity, but if obesity is followed by a cognitive decline, the ability to process emotions could also decline. Persons with severe cognitive deficiencies were however excluded. The link between physical inactivity and obesity, which probably is two-ways, is in accordance with previous research in non-diabetic populations [39, 40]. The importance of obesity in T1DM in this study was demonstrated by the strong association between abdominal obesity and cardiovascular complications, as shown in previous research [1, 2, 8]. We found a gender difference with a higher obesity prevalence in the women than in the men, which also has been shown previously [10].

By this study, a new target in diabetes care emerges, “increased emotional awareness”, which today is not considered in conventional diabetes management. Persons with alexithymia have difficulties distinguishing bodily sensations due to emotional arousal from bodily sensations of somatic origin [14]. Difficulty identifying feelings might thus make it difficult to distinguish hunger from emotions involved in stress, anxiety or boredom, factors which serve as triggers for emotional eating [12]. One issue connected to diabetes emotional distress is that the affected persons do not know whether their mood and feelings are related to their diabetes or not [41]. This is probably particularly difficult for persons with alexithymia. The link between alexithymia and obesity might be one explanatory factor for the increased cardiovascular mortality found in men with alexithymia [18].

Treatment with a combination of multiple daily insulin injections and oral antidiabetic agents was associated with abdominal obesity in this study. Oral antidiabetic agents should however not be regarded as a cause of obesity, as they were added to insulin with the aim of reducing obesity and insulin resistance.

The women seemed to have more psychological symptoms than men, with higher prevalence of anxiety, high self-hate and self-blame, and low self-affirmation and self-love which also is in accordance with previous research [27]. However, these factors were not associated with abdominal obesity. The use of antidepressants was associated with abdominal obesity in women but not in men, though the frequency of antidepressant use did not differ between men and women. To adequately analyse why antidepressants were associated with obesity in women only, we would have needed to know whether women were prescribed different types of antidepressants than men, the prescribed daily dose, and the duration of medication.

There are several subjects for future research. The mechanism for the demonstrated higher obesity prevalence in women, compared both to the men with T1DM and to women in the general population, was not explained by this study and further research of this subject is necessary. Further, it would be of interest to explore whether alexithymia and diabetes emotional distress is associated, which to our knowledge has not been studied. Finally, we propose that interventions focusing on increased emotional awareness should be performed and evaluated for persons with T1DM, obesity and alexithymia [31, 32, 42–44].

## Conclusions

Abdominal obesity in T1DM was associated with women, alexithymia, particularly the alexithymia subfactor “difficulty identifying feelings”, and physical inactivity. As abdominal obesity is detrimental in diabetes due to its association with cardiovascular complications, our results suggest two risk factor treatment targets: increased emotional awareness and increased physical activity.

## Abbreviations

AOR: Adjusted odds ratio
BMI: Body mass index
CI: Confidence interval
COR: Crude odds ratio
CSII: Continuous subcutaneous insulin infusion
DDF: Difficulty describing feelings
DIF: Difficulty identifying feelings
EOT: Externally oriented thinking
HADS-A: Hospital Anxiety and Depression scale-anxiety-anxiety subscale
HADS-D: Hospital Anxiety and Depression Scale-depression subscale
MDII: Multiple daily insulin injections
OAA: Oral antidiabetic agents
SASB: Structural Analysis of Social Behavior assessment tool
T1DM: Type 1 diabetes mellitus
TAS-20: Toronto Alexithymia Scale-20 items
WC: Waist circumference
